# Isolated right atrial appendage (RAA) rupture in blunt trauma – a case report and an anatomic study comparing RAA and right atrium (RA) wall thickness

**DOI:** 10.1186/1749-7922-2-5

**Published:** 2007-02-15

**Authors:** Adoniram M Figueiredo, Renato S Poggetti, Fabio G Quintavalle, Belchor Fontes, Moise Dalva, Riad N Younes, Fabio B Jatene, Dario Birolini

**Affiliations:** 1 Emergency Surgery Service, Clinics Hospital, University of São Paulo Medical School, Rua Dr. Eneas de C Aguiar, 255 CEP 05403-000, São Paulo (SP), Brazil; 2 Heart Institute (INCOR), Clinics Hospital, University of São Paulo Medical School, Rua Dr. Eneas de C Aguiar, 255 CEP 05403-000, São Paulo (SP), Brazil; 3 Trauma Discipline, Department of Surgery, University of São Paulo Medical School, Rua Dr. Eneas de C Aguiar, 255 CEP 05403-000, São Paulo (SP), Brazil

## Abstract

**Background:**

Heart chambers rupture in blunt trauma is uncommon and is associated with a high mortality. The determinant factors, and the incidence of isolated heart chambers rupture remains undetermined. Isolated rupture of the right atrium appendage (RAA) is very rare, with 8 cases reported in the reviewed literature. The thin wall of the RAA has been presumed to render this chamber more prone to rupture in blunt trauma.

**Objective:**

To report a case of isolated RAA rupture in blunt trauma, and to compare right atrium (RA) and RAA wall thickness in a necropsy study.

**Methods:**

The thickness of RA and RAA wall of hearts from cadavers of fatal penetrating head trauma victims was measured. Our case of isolated RAA rupture is presented. The main findings of the 8 cases reported in the literature, and the findings of our case, were organized in a table.

**Result:**

The comparison of the data showed that wall thickness of the RAA (0.53 ± 0.33 mm) was significantly thinner than that of RA (1.11 ± 0.42 mm) (p < 0.05).

**Comments:**

In all these 9 cases of isolated RAA rupture, cardiac tamponade occurred, RAA rupture was diagnosed intraoperatively and sutured, and the patients survived. Main mechanisms hypothesized for heart chamber rupture include mechanical compression coincident with phases of cardiac cycle, leading to high hydrostatic pressure inside the chamber. Published series include numerous cases of RA rupture, and only a few cases of RAA rupture.

**Conclusion:**

Thus, our data suggests that wall thickness is not a determinant factor for RA or RAA rupture in blunt trauma.

## Background

Traumatic rupture of the heart occurs in 0.5% blunt trauma (BT) [1] cases, with a usually difficult diagnosis, and presents 75% [2] and 81.3% [3] reported mortality rates when heart chambers rupture occurs. Although all four heart chambers were considered having an equal chance of rupture in BT [4], ventricular tears tend to be rapidly fatal and few patients survive [5], while there is little debate that the majority of survivors of blunt cardiac rupture are those with atrial rupture [2,6-12]. However, the real incidence of isolated rupture of heart chambers in BT remains unknown. Isolated rupture of RAA in BT is rare, with eight cases reported in the reviewed literature (see Table 1) [9,12-17].

**Table 1:** Common diagnostic findings and therapeutic procedures reported in the literature (cases 1–8) and the present case report (case 9)

**FINDINGS**	**THE CASES**
**Hemodynamics**	
CVP ≥ 20 H_2_O	1, 3, 9
SBP ≤ 70 mmHg	2, 3, 4, 5, 6, 9
**Thorax**	
Bruised chest wall	2
Pneumothorax	4
Lung contusion	4
Sternal fracture	2, 6, 7
Cyanosis	1, 6
Muffled heart sounds	1, 2, 3, 4, 9
Enlarged heart silluette	4, 5, 6
Widened mediastinum	4, 5,,6, 9
Cardiac tamponade	1, 2, 3, 4, 5, 6, 7, 9
Pericardial tear associated to RAA injury	3
Hemothorax	3
Rib fracture	7, 8
**Neck**	
Neck vein distension	2, 5, 6
**Abdomen**	
Liver injury	1, 2, 4, 8, 9
Spleen injury	4, 8, 9
**Extremity**	
Lower limb fractures	1, 4, 7, 8, 9
**SURGICAL PROCEDURES**	
Pericardiocentesis	1, 3, 8
Laparotomy	1. 4. 5.7, 8, 9
Sub-xiphoidal pericardial window	4, 5, 7, 9
Opening of the diaphragm at laparotomy	3
Access to RAA:	
Bilateral thoracotomy	9
Medial sternotomy	1, 2, 3, 4, 5, 6, 7, 8
RAA injury repair:	
Ligature	2
Suture	1, 3, 4, 5, 6,7, 8, 9

Main diagnostic and surgical findings and/or procedures performed in the 9 cases of isolated blunt traumatic rupture of the right atrium appendage (RAA) here discussed CVP: central venous pressue. SBP: systolic blood pressure. RAA: right atrium appendage. Figures in the right column of the Table represent the cases number: 1 (Trueblood et al [5]), 2 (Galton et al [6]), 3 (Leavitt et al [7]), 4 and 5 (Kupferschmidt et al [8]), 6 (Degiannis E et al [9]), 7 (Dagenais F et al [10]), 8 (Ilkjoer LB et al [11]).

Among the diverse mechanisms proposed for traumatic rupture of heart chambers [8,9,12,13], the thickness of the chamber wall has been presumed to be a possible factor [12,13], and the atrium appendage has been supposed to be the thinnest and weakest portion of the heart [13]. However, no research has been mentioned supporting this hypothesis. Furthermore, no anatomic study has been reported comparing RAA and RA wall thickness as possible determinant factor for their susceptibility to rupture. Therefore, the **objectives **of this study were: 1) To compare the thickness of the RAA and RA walls of hearts of lethal head trauma victims; 2) to present a case report of isolated BT rupture of the RAA, and discuss the role of wall thickness as a possible factor.

## A comparative evaluation of the RAA and RA wall thickness

### Method

To evaluate the wall thickness of RAA and RA of trauma victims, twenty-eight hearts that had been removed during autopsy, at the Legal Medicine Institute of the USPSM, from cadavers of penetrating lethal head trauma victims aged from 14 to 84 (mean 31) years, were studied. The hearts were preserved in formaldehyde for anatomic study at the MAPD of the INCOR, CH-USPSM. The study was performed according to the norms of the Ethics Committee of the institution. With the use of a pachymeter, the RAA wall thickness was measured at the apex of this chamber. The RA wall thickness was measured at the antero-superior wall of the RA, at 1 cm from the base of the RAA. The data obtained for the RA and RAA wall thickness (mm) were expressed as mean ± SEM, and compared using Student's t test. P values < 0.05 were considered significant.

## Results

The wall thickness obtained for the RA was 1.11 ± 0.42 mm, and for the RAA was 0.53 ± 0.33 mm (Figure), and the comparison of these results showed that RAA thickness was significantly smaller than that of RA (p < 0.05).

## Case report

A 16-year old male, unrestrained passenger was a victim of high speed frontal collision of the car with an electricity pole, and 30 min thereafter was admitted to the emergency room (ER). On admission, he was complaining of thoracic and abdominal pain, and presented free airways, normal chest expansion, BP:70 × 40 mmHg, HR: 120 bpm, RR: 18 epm, GCS: 15, RTS: 6.4, abdominal tenderness, and a closed right femur fracture. Plain X ray films suggested a widened mediastinum. After 3000 mL of warmed saline infusion, his BP was 60 × 30 mmHg, and his HR was 120 bpm. A diagnostic peritoneal lavage was positive for blood. As ultrasonography was unavailable, a laparotomy was performed, with splenectomy and cauterization of a superficial liver injury. After infusion of 6000 more mL saline, 8 units PRBC, and 9 units fresh frozen plasma, the patient's conditions worsened, with muffled heart sounds, undetectable peripheral pulses, and CVP of 20 cmH_2_0. Cardiac tamponade was suspected, and confirmed through a subxiphoid pericardial window. A thoracotomy was performed, with pericardiotomy and removal of blood clots from the pericardial cavity. A 0.5 cm bleeding tear in the apex of the RAA was found, and closed with sutures. The thoracotomy incision was closed. The patient was discharged from the hospital on the 20th postoperative day. His injury severity score (ISS) was 50, and his TRISSCAN was 0.31.

The main data of this case are presented (see Table 1) together with the main data of the 8 cases (cases 1 through 8) of isolated RAA rupture reported in the literature [9,12-17]. Eleven other RAA cases mentioned in the literature without clear informative data are not included here [8,13].

The 9 cases (see Table) comprised 5 men and 4 women, aged 15 to 34 (mean: 28) years, all involved in high speed MVC (8 cars and 1 motorcycle). The victims were a motorcyclist (case 1), unrestrained car drivers (cases 2, 3, 4, 5, 8), or passengers (cases 7 and 9), or unspecified position (case 6). Overall, the main diagnostic findings in these 9 cases included hypotension or persistent unresponsive shock (cases 4, 5, 8, 9), muffled heart sounds (cases 1, 2, 3, 4, 9), high CVP values (cases 1, 3, 9), neck vein distension (cases 2, 5, 6), thoracic imaging alterations (cases 2, 4, 5, 6, 9), and traumatic ascitis (case 7). Cardiac tamponade was present in the 9 cases. All the patients underwent a thoracotomy allowing RAA injury identification and repair, and all patients survived. The length of hospital stay ranged from 8 to 20 days.

## Discussion

Blunt trauma rupture of heart chambers is more frequent in MVC with great deceleration leading to high energy impact of drivers' thorax on the steering wheel [18-20]. In the care of these patients, the most frequent findings of cardiac rupture may be identified on clinical and roentnographic evaluation (see Table) [2,8]. However, the identification of which heart chamber is ruptured has been invariably achieved intraoperatively [21], as it was in these 9 cases. The diagnostic findings and surgical procedures, observed in our case, are common to most of the other reported cases (see Table). Although in all these 9 cases the patients survived, diverse cases of atrial appendage rupture have been found in fatal BT victims, but the mortality of isolated RAA rupture has not been discussed, and remains undetermined.

### Mechanisms of traumatic heart rupture

Although not supported by clinical or experimental data, diverse mechanisms were proposed for heart rupture in BT, including: sudden deceleration [12], the occurrence of rib fractures [5,11,14,22,23] increased intracardiac hydraulic pressure resulting from abdominal/lower limb venous compression [12], mechanical heart compression [8,12,13], the phase of the cardiac cycle [9,24], concommitant interaction of these factors [9]. In the analysis of the here discussed 9 cases, none of the above mentioned factors could be identified as the main determinant of the RAA rupture.

According to some authors, ventricular versus atrial rupture depends on the phase of the cardiac cycle, atrium rupture being more likely to occur under a forceful compression in late systole, when the atria are most distended with venous blood, and the atrioventricular valves are closed [11,24]. Given the anatomicotopographic relationship between the RAA and the RA, their walls are under the same hydraulic pressure. According to Trueblood [13], the thinner wall of the RAA could render this chamber more prone to rupture under heart compression between the sternum and thoracic spine. The results of our anatomic study show that RAA wall is thinner than RA wall. Therefore, under these circumstances, RAA rupture would be expected to occur more frequently than RA rupture. However, the incidence of RA rupture reported by diverse authors [10,11,22], is higher than that of RAA rupture [8,9,12-17]. Thus, other factors than wall thickness may be involved in the mechanism of isolated RAA rupture in blunt trauma.

In **conclusion**, the findings of our study indicate that the RAA wall is thinner than that of the RA. However, given the reported higher incidence of RA rupture than of RAA rupture, our findings do not suggest wall thickness as a predominant factor either for RA or RAA rupture in blunt trauma.

## Competing interests

Concerning the present manuscript, the authors declare that there are no personal, or financial, or non-financial competing influence, interests, or conflicts involving them with other people or organizations.
